# Enterprise internal audit data encryption based on blockchain technology

**DOI:** 10.1371/journal.pone.0315759

**Published:** 2025-01-10

**Authors:** Lixia Gao

**Affiliations:** Taiyuan University, Taiyuan, China; Jazan University, SAUDI ARABIA

## Abstract

Internal auditing demands innovative and secure solutions in today’s business environment, with increasing competitive pressure and frequent occurrences of risky and illegal behaviours. Blockchain along with secure databases like encryption improves internal audit security through immutability and transparency. Hence integrating blockchain with homomorphic encryption and multi-factor authentication improves privacy and mitigates computational overhead. Recently, blockchain applications for internal audits in the enterprise sector are still emerging. Thus, blockchain technology in auditing provides the benefits of enhanced transparency and immutability in data processing, which can establish new solutions for internal auditing but still lacks encryption techniques. The research proposed a framework called “BlockCryptoAudit” to enhance internal audit processes through cryptographic encryption methods and blockchain technology, ensuring secure and transparent audit operations. The proposed approach integrates an additive homomorphic Paillier encryption scheme with blockchain to create a safe and tamper-resident audit trail. Utilizing homomorphic Paillier encryption, BlockCryptoAudit ensures that computations may be performed on encrypted audit data while safeguarding data privacy. The applied blockchain hyperledger component guarantees the immutability and transparency of encrypted audit records, resulting in a decentralized and tamper-resistant record. By limiting data accessibility to authorized individuals based on specified responsibilities, role-based access restrictions handled using smart contracts further strengthen security. The study protects audit data’s security and confidentiality by encrypting it and putting it on a blockchain. The study compares the proposed BlockCryptoAudit with models like B-OAP, BSE-DF, and EG-FLB regarding risk mitigation, audit quality, security overhead, and audit trail effectiveness. With little security overhead, BlockCryptoAudit beats out B-OAP, BSE-DF, and EG-FLB in terms of risk mitigation (98%) and audit quality (99%). It is an effective way to improve internal audit processes and guarantee data integrity due to its high performance.

## 1. Introduction

An effective internal audit and robust enterprise risk management are crucial for enhancing corporate governance, driving adequate audit planning execution, and boosting financial data performance [1]. The audit process is being impacted by concerns about the dependability, completeness, integrity, and security arising from the growing data and technological advances. Internal auditing is an independent, objective activity that enhances operations by systematically improving risk management, control, and governance [2]. Traditional auditing methods were generally performed through internal investigation for threat and risk assessment in daily enterprise operations processes. Hence, blockchain implementation is suggested in the audit management system to support more secure and sustainable business operations [3]. The study shows that blockchain technology significantly improves the reliability of financial data confidentiality, highlighting its potential to enhance data security systems in economic enterprises involved with internal audit data [4]. Implementing blockchain in enterprises’ accounting and auditing highlights its potential to improve system reliability and mitigate fraudulent activities and errors in records related to internal audits [5].

The urgency of enhanced cybersecurity measures against data breaches and cyber threats, with the exploration of blockchain technologies for data protection, secures audit data through encryption in enterprises [6]. A compressive integrity auditing protocol has been designed to ensure data integrity with reduced communication overheads using strong homomorphic encryption for remote integrity auditing protocols [7]. The research investigated the influence of blockchain adoption on the internal audit function and its role in blockchain smart contracts within the financial sector. The study addressed the knowledge gap by examining the substantial benefits and challenges of integrating blockchain technology into modern accounting systems [8]. Likewise, the survey of applying data encryption mechanisms in personal data protection complements and has provided the users with the control key for accessing the records. Internal audit research demonstrates that the blockchain’s more comprehensive applications in data security and user control are essential in securing enterprise internal audit data [9]. For these security reasons, the study employed an audit and assurance value chain to categorize the audit process into access, verify, and protect data and access controls [10].

They focused on transaction security using blockchain mechanisms to enhance privacy data security in international trade. The results efficiently reduced data anomalies and trade transactions among data audits and backups in enterprises [11] for secure data sharing and exchange between audit authority control services and the blockchain-based hierarchical management for encrypted storage combined with ciphertext retrieval [12]. The encrypted data can be stored in a distributed fashion using blockchain technology. It eliminates the traditional way of centrally storing services to provide secure role-based access control [13]. The study of Blockchain technology addresses its potential effects on audit processes, including preparation, collection of audit evidence, and, ultimately, application of expert judgement to the accounting records [14]. The evaluation data considers client importance, names, audit opinions, and lags for measuring the audit efficiency [15]. Thus, the access authority-based security feature in the service application must be decentralized with an appropriate detection rate [16]. The internal audit team oversees, assesses, and optimizes the organization’s risk of business operations, control, and corporate governance to accomplish internal auditing under the supervision of the enterprise development strategy [17]. While previous studies have focused on using blockchain for audit trail integrity and fraud detection, less attention has been given to developing robust encryption frameworks that can efficiently handle vast volumes of audit data while ensuring risk factors in compliance and data privacy requirements. Secure databases and enhanced encryption can increase internal audit security and transparency with blockchain. The immutability, transparency, and decentralized access control of blockchain make it a helpful tool for improving internal audit data security. Blockchain technology can calculate encrypted data with cryptographic approaches like homomorphic encryption, increasing privacy and auditability. Blockchain ensures decentralized data storage and immutability. Blockchain solutions like cloud-based encrypted systems with multi-factor authentication and robust encryption methods for safe audit trails are the most secure. These options avoid blockchain computing overhead.

Given the increasing fraudulent behaviours and data security concerns in modern business environments, the study addresses the need for more secure and reliable internal audit processes in enterprises. By addressing these challenges, crypto features integrated with blockchain in enterprise internal audit operations can act as a deterrent, reducing the probability of illegal behaviour and ensuring more secure and reliable internal audit data encryption. The key novelty of the proposed BlockCryptoAudit lies in integrating the additive homomorphic property of Paillier encryption with blockchain technology to enhance the internal audit data process.

Regarding internal audits, BlockCryptoAudit is a game-changer since it combines blockchain technology with additive homomorphic Paillier encryption in an innovative manner. Within the context of risk assessments and other auditing duties, this technology enables secure computations on encrypted audit data, safeguarding client information’s confidentiality. Blockchain technology’s immutability and transparency ensure that audit records are tamper-proof and visible. Smart contracts enforce role-based access control, which restricts data access based on user roles. BlockCryptoAudit stands out from other models due to its automatic audit trail, strengthening the framework by capturing all audit actions comprehensively and tamper-proof. This characteristic makes BlockCryptoAudit stand out from other models.

The model allows for secure computation and storage of sensitive audit data while maintaining the ability to perform risk assessments and other audit functions on encrypted data. The role-based access control and automated audit trail further enhance the novelty of the proposed system in the context of enterprise internal audits.

Internal auditing solutions like digital and traditional auditing tools often fail to ensure transparency, preserve data, and discover fraud. Even though many systems use traditional encryption, audit trails are vulnerable to internal and external threats. These methods cannot prevent data tampering or illegal manipulation. Single points of failure in centralized audit systems increase data breaches and lower audit integrity confidence.

Although there are several ways to prevent illegal and harmful internal audits, the current knowledge base is lacking. B-OAP and BSE-DF, blockchain-based platforms, are immutable and transparent but have scaling issues and need expensive computer resources. These methods lack strong encryption, leaving privileged audit data exposed. Advanced Encryption Standard (AES) and elliptic curve algorithm-based encryption systems give stronger security, however they lack complete audit trails, making fraud detection harder. Common auditing methods that involve human monitoring and centralised data storage are vulnerable to data tampering and agency attacks. Although these technologies have made progress in risk detection and access control, they are still far from being able to manage massive datasets, provide secure real-time monitoring, and provide audit procedures that are impossible to manipulate. Addressing these issues requires more complete solutions with scalability, audit trail integrity, and encryption.

BlockCryptoAudit is a framework that stands out owing to its unique integration and execution. However, the concept of utilizing blockchain technology and homomorphic encryption for data auditing is not a novel one. Since it combines blockchain technology with additive homomorphic Paillier encryption, the BlockCryptoAudit method is a novel approach. This combination makes it possible to do risk assessments and other operations on encrypted data and compute and securely store audit data. Additionally, it enables the execution of these processes. Furthermore, in addition to this connectivity, there is an automated audit trail and access control based on roles through intelligent contracts. The combination of these traits results in an increased level of security, transparency, and resistance to tampering with the system. The drawbacks of existing models are addressed by this all-encompassing solution, which ensures robust data protection and audit records that are full and cannot be altered.

The significant contribution of the article include.

(1) BlockCrypto Audit uses blockchain technology and additive homomorphic Paillier encryption to manipulate audit data while securely maintaining its secrecy and integrity. This connection covers critical auditing system gaps in data security and fraud detection.

(2) The architecture improves internal audits by ensuring tamper-proof records and automating audit trails. This enhances audit reliability and efficacy by allowing real-time data evaluation and monitoring to detect unsafe activities and ensure compliance.

(3) BlockCrypto Audit significantly minimizes data integrity and unauthorized access risks compared to other approaches. Scalable performance allows it to manage massive audit data and several nodes without security overhead, making it adaptable to varied organizational contexts.

The reminder sections of the research work are discussed as follows: Section 2 discusses a related works on the various approaches to enterprise internal auditing is discussed. Section 3 discusses the BlockCryptoAudit layout and each phase of the audit process using open-source dataset. Experimental evaluation and comparative analysis with an existing model are discussed in Section 4 using the metrics risk mitigation, audit quality, security overhead, audit trail effectiveness using computation cost, storage overhead and energy consumption. Section 5 discusses the research conclusion and its remarks.

## 2. Related works

### 2.1 Case studies related to internal audits of enterprises

Li and Bai [18] offered a Blockchain-based Online Audit Platform (B-OAP) with four modules: audit access, auditee primary data, blockchain auditing application service, and blockchain technical support to analyze the work of Zhiyuan Technology Company integrating blockchain into online auditing. The study shows that processes have been simplified, and data integrity has been enhanced using Zhiyuan’s audit data. A blockchain audits access module handles the public key encryption, and operational levels are encrypted using asymmetric key encryption. There are still obstacles, such as inaccurate data transfer and thorough data analysis. Dong and Pan [19] employed a questionnaire-based approach to analyze audit cases of enterprises in Chinese and one in Australia. This study examined the effects of blockchain technology on audit risk using the Synchronous Automatic Ledgers (SAL) methodology. Blockchain technology raises both inherent and control risks, according to the 28-person study, due to the lack of expertise in business audits. This, in turn, makes auditing more difficult and increases the demand for auditors’ ethical and professional competence. The results have shortcomings, such as insufficient study samples and the absence of expert involvement in questionnaire development.

Problems with limited experts like auditors, excessive workloads, and disjointed data support were resolved by Si [20] and enhanced the data quality and audit efficiency. The study used multisystem data management, K-means clustering, and various decision tree algorithms like Fuzzy-based ID3, Min-Ambiguity, C4.5, and CART. Fuzzy ID3 outperformed the other algorithms in terms of precision in classification and rule development. The study may not be generalizable to fewer or smaller data-intensive businesses due to its emphasis on large, data-rich corporations.

### 2.2 Blockchain’s potential in internal audits

Parmoodeh et al. [21] applied the potential of blockchain technology in auditing by analyzing semi-structured interviews conducted with professionals from Big Four and non-Big Four businesses. The three primary topics identified are audit practices, processes, and obstacles to adopting blockchain. It also shows how BCT’s immutable audit record and lack of tampering might drastically cut fraud detection costs. There is currently no standardized structure for the deployment of blockchain, and there are difficulties in adopting it generally across different audit enterprises. Zidan et al. [22] built a Blockchain-based Strong Encryption and Dynamic Framework (BSE-DF) to investigate the potential of blockchain technology for enhancing enterprise cybersecurity. The study shows that auditing processes with blockchain significantly protect sensitive firm data and make the system more responsive to employee fraud. Dataset features such as records of transactions, and security logs demonstrate how blockchain outperforms conventional systems in risk mitigation. Thoughtful access controls and frequent monitoring are crucial to blockchain’s success. One limitation is that proper employee training is necessary to utilize blockchain technology fully, as are appropriate regulation policies.

Liu et al. [23] suggested a novel audit method that applied blockchain technology to improve data integrity in shared environments with storage security. The model presents a Signature (Sig) mechanism that is both lightweight and efficient, allowing for Member Revocation (MR) without sharing source data. Performance evaluations show how efficient the method is, while security analyses confirm its reliability. The difficulty of using blockchain technology and possible scalability problems in large-scale deployments are two limitations.

Sun et al. [24] proposed an audit scheme for Encrypted Gradients in Federated Learning built on the Blockchain (EG-FLB). This will help prevent data poisoning and free-riding attacks in audit procedures. It ensures privacy and accountability using different blockchains for gradient gathering and aggregation. Experimental results and security analyses demonstrate that harmful activity improves the security of federated learning. However, there are obstacles, such as the computational load of homomorphic data encryption and blockchain scalability.

The existing study in Table 1 reveals substantial breakthroughs and applications of blockchain technology for enhancing audit processes. Nevertheless, there is still a significant lack of research regarding combining blockchain technology with modern encryption methods to solve scalability problems and improve data privacy in audit situations. Developing robust encryption frameworks that can efficiently manage massive amounts of audit data while guaranteeing compliance with data privacy requirements has received less attention than using blockchain for audit trail integrity and identifying fraudulent/ risky transactions, which has been the subject of multiple studies. In addition, the difficulties in scaling homomorphic encryption in blockchain systems prevent its broad use in auditing techniques. This calls for more excellent investigation into cryptographic protocols to ensure audit data’s security and scalability. If blockchain technology is to be widely and effectively used to improve audit integrity and security in various enterprise scenarios, these gaps must be filled.

**Table 1:** Literature survey.

Author	Method	Application	Advantages	Limitations
Wang et al. [25]	DEA effectiveness model and entropy weight approach	Examine the blockchain business asset management mode.	An efficient blockchain-based business asset management accounting solution with realistic development goals	Scalability issues and High computational cost
Khellaf et al. [26]	Mobile Ecosystem management tools	Secure mobile host-enterprise system interactions	Improves Mobile Application Management security and efficiency, allowing policy management flexibility.	Blockchain’s scaling and the difficulties of integrating mobile agents into infrastructure
Alkhatib et al. [27]	Elliptic curve digital signature and AES encryption (AES_ECCDSA)	A secure and efficient Ethereum blockchain-based academic QAS.	Automates quality transactions, improves information governance, lowers operational costs, and secures application layers.	More extensive data takes longer and costs more in blockchain processes.
Sarram et al. [28]	Cross-sectional and structural equation modelling	Blockchain technology boosts confidence and data reliability for Jordanian SMEs’ external audits.	The study concluded that blockchain qualities boost audit quality by enhancing transparency, accuracy, and confidence.	An emphasis on Jordanian SMEs may limit its application to other industries.

Current internal auditing solutions, like traditional methods and digital tools, faces challenges with transparency, data preservation, and fraud detection. These are vulnerable to data tempering, lack complete audit trails, and are prone to compromise due to centralized approach. Blockchain platforms like B-OAP and BSE-DF provides immutability but suffer from scaling problem and weak encryption method. Though, the existing techniques are strong fail to ensure full audit trails. These existing research gaps highlights the need for scalable, secure solutions with comprehensive audit trails, which the proposed BlockCryptoAudit aims to address these challenges.

## 3. Research methodology

**Fig 1:**
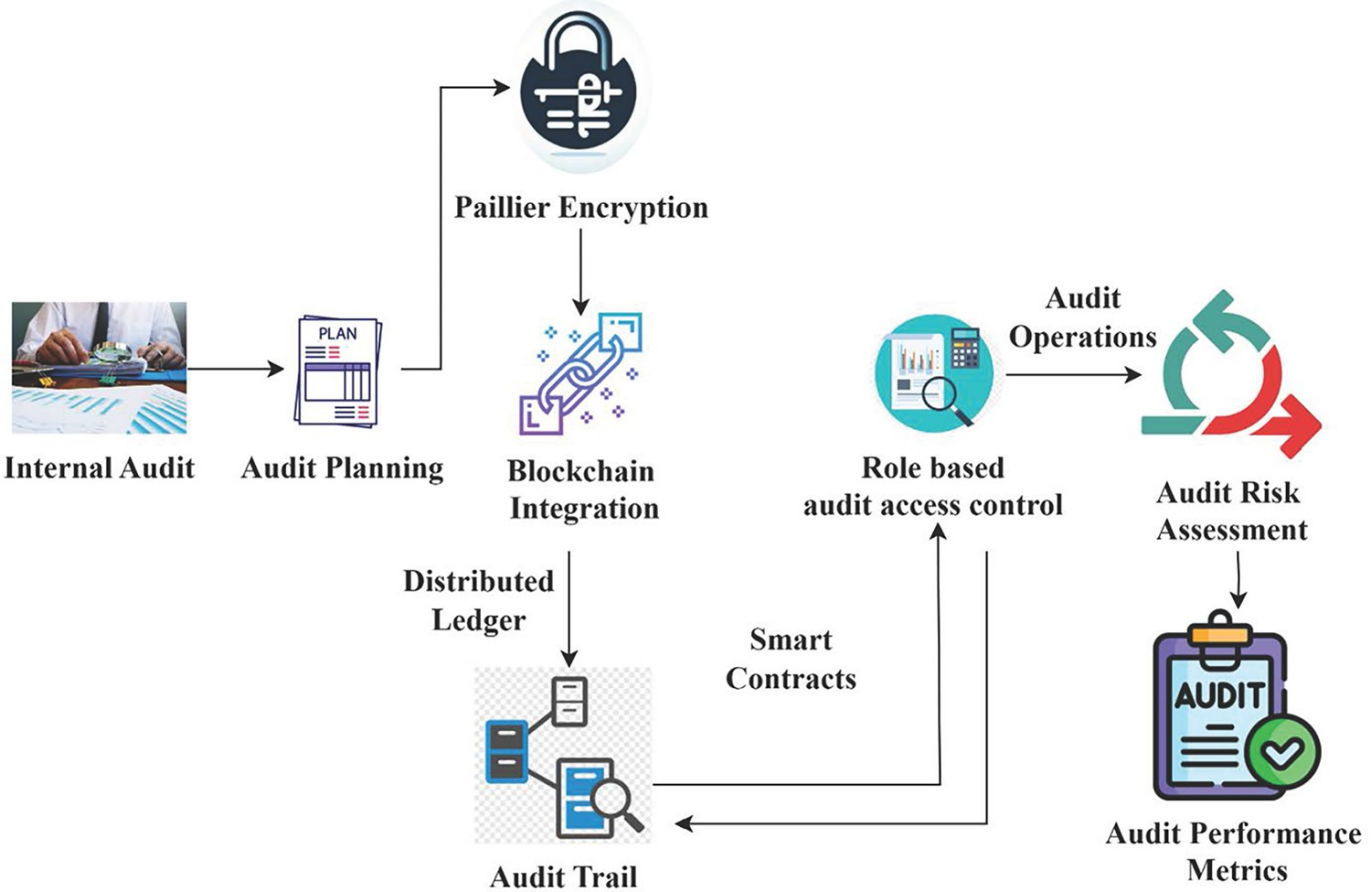
BlockCryptoAudit layout for enterprise internal audit.

BlockCryptoAudit aims to promote secure audit methods in many industries by highlighting blockchain’s built-in security features and scalable encryption solutions. An internal audit data security relies heavily on the critical generation method employed by Paillier encryption, which lays a solid cryptographic groundwork. The security of audit data held on distributed ledgers is enhanced by integrating Paillier encryption with the blockchain system. Secure encryption of audit logs, financial records, and other sensitive data prevents their unlawful access or manipulation. Paillier’s encryption powers are enhanced by the immutability and transparency of blockchain, which creates an audit trail that cannot be tampered with. This systematic strategy guarantees conformity with internal audit goals and procedures while integrating blockchain and Paillier encryption to secure data. Confidentiality of internal audit data is preserved using Paillier encryption keys generated from substantial prime numbers. Data protection rules and company security policies make this a must-have. Secure calculations on encrypted data are made possible by the homomorphic features of Paillier encryption. It helps with audit processes such as risk aggregating, fraud detection, and compliance monitoring, all while keeping data privacy secure. Accountability is improved, and audit standard compliance is made easier with automated audit trail reporting. By providing a secure, efficient, and scalable architecture that meets the ever-changing demands of auditing data integrity and privacy, BlockCryptoAudit intends to change audit processes significantly, as described in Fig 1.

### 3.1 Data preparation

The fundamental functionalities blockchain technology provides for preparing data is to ensure the security and suppression of fraud throughout audits. Cryptography, real-time transaction publishing, and hosting intelligent contracts are all included in the audit operations. On top of gathering and analyzing company data, auditors also search historically unavailable industry datasets- factors that influence a company’s accounting and internal control systems, as well as the processing, storage, and communication of financial data. Internal auditors can speedily review data sets, look for secure patterns, and discover novel relationships between data points that may otherwise go unnoticed, all while conducting audits inside an organization.

From the input data attributes, the money value *m*_*vl*_ is considered for analyzing financial metrics and the risk factors *R*_*A*_ and *R*_*B*_ for different audit criteria A and B. Internal audit operations may include recording a transaction, verifying a piece of data, performing a risk assessment calculation, generating an audit report, and accessing encrypted audit records. The system can access the audit performance under different risk metrics by varying the number of operations.

### 3.2 Audit planning

Develop an audit plan and establish requirements that influence the audit efficiency and results. For the BlockCryptoAudit framework, audit objectives include assessing the effectiveness of the blockchain-based audit trail in maintaining data integrity. Evaluating the security of encrypted data stored on the blockchain, measuring the efficiency of audit processes using the new system, verifying compliance with relevant regulations and internal control policies, and identifying the potential areas for improvement in the audit process. These objectives guide the entire audit process, determining the evidence regarding money value, history of risk and other financial risk factors. In addition to these objectives, timelines and responsibilities among team members of the audit group need to be considered. To learn how audit team members find, evaluate, and rank organizational risks and how they incorporate this data into audit plans, it’s a good idea to conduct meetings with them. With this knowledge, an internal audit planning structure for risk assessment decisions in audit enterprises can be more effectively put forth.

By analyzing historical risk *H*_*R*_, auditors assess the cumulative risk posed by past audit findings or events. Highlighting locations with considerable max(H) and exposure that may require more robust controls informs audit planning. Organizations can prioritize interventions and resources according to trends in historical data and hazards by incorporating *H*_*i*_ into risk management frameworks. Organizational resilience is improved, and this proactive approach fortifies risk mitigation methods.

$$H_{R}=\sum_{i=1}^{n}{\left(\frac{H_{i},}{max(H)}\right)}^{2}\times R_{F_{i}}$$ (1)

In Eq (1), $R_{F_{i}}$ defines the risk factors associated with each historical data point *H*_*i*_, quantifying the risk level and impact attributed to each historical factor. The maximum value among all historical data points, *H*_*i*_, is a normalized *max*(*H*). For planning an audit and risk management, this cumulative assessment of risks sheds light on the historical risk exposure and its effect. Auditors use *H*_*R*_ to calculate the total risk caused by audit results or occurrences in the past. Drawing attention to places with high levels of historical risk exposure helps with audit planning by indicating which ones could need more thorough investigation or better controls from the integrated approach.

When planning internal audits, auditors consult substantial risk assessment data. It is necessary to comprehend distinct risk variables, historical data, and control contexts to address the most severe risks. Risk scores and categories are part of the internal audit data set that auditors use to find possible risky spots due to a lack of data security. By using this data-driven strategy, internal audits can zero in on the areas that might have the biggest influence on the goals and controls of the business.

### 3.3 Internal audit data encryption with Paillier algorithm

The general representation of Paillier encryption in the internal audit data is to sum up two encrypted values of plaintext messages *m*1 and *m*2; these could represent sensitive audit data such as financial transactions, audit reports and access logs. With the help of Paillier encryption, the parameter *en*(*m*1) and *en*(*m*2) can be computed as:

$$en(m1+m2)=en(m1)∙en(m2)mod\;n^{2}$$ (2)

The operation described in Eq (2) performs the computation of the product of encrypted values *en*(*m*1) and *en*(*m*2), where 0≤*m*<*n*. This property enables operations on encrypted data without decrypting, which is critical for preserving data confidentiality during computations. The variable *n* defines the product of two large primes ℘_1_ and ℘_2_ in the Paillier cryptosystem. These variables generate public and private keys for encryption and decryption operations. The size of *n* directly impacts the security and efficiency of the encryption scheme.

Key Generation:

Generate Paillier encryption keys for choosing two large prime numbers ℘_1_ and ℘_2_ and ensures that *n* is sufficiently large, making it computationally feasible to factorize *n* and derive ℘_1_ and ℘_2_ from it to computer modulus and given as:

$${n=℘}_{1}{\times ℘}_{2}$$ (3)

Eq (3), with the selection of large prime numbers, provides a robust foundation for encrypting sensitive data attributes such as financial metrics like money value ℳ_*vl*_, inherent risk as *in*_*R*_ and *ctrl*_*R*_ for indicating compliance in an enterprise.

Then select a random integer *g* that indicates the multiplicative group modulo *n*^2^, and facilitates the additive homomorphic property of Paillier encryption. This feature allows audit data metrics to be safely aggregated on the blockchain without compromising data privacy. For this research, an encryption of money value *m*_*vl*_ of plain text *m* is computed using Eq (4),

$$en(M_{vl})=g^{m_{vl}}∙r^{n}\;mod\;n^{2}$$ (4)

Where $r\in Z_{n}^{*}$ indicates that *r* is a random integer with the range of 0<*r*<*n*, and belongs to a non-zero integers modulo *n*. The public key allocated are *n*, and *g*, followed by storage on the blockchain, is given as *en*(*m*_*vl*_). For decrypting the encrypted money value noted as *en*(ℳ_*vl*_) the computation proceeds as follows using Eq (5) and Eq (6),

$$de({\zeta (M}_{vl}))=(({\left({en(M}_{vl}\right)}^{\varphi }\;mod\;n^{2})∙\mu )mod\;n$$ (5)

$$\varphi =lcm(({℘}_{1}-1)({℘}_{2}-1))$$ (6)

The variable *φ* defines the Carmichael function $lcm(({℘}_{1}-1)({℘}_{2}-1))$ derived from the prime factors ℘_1_ and ℘_2_ of the modulus *n*. The term *μ* defines the modular inverse of $L({\left({en(M}_{vl}\right)}^{\varphi }\;mod\;n^{2})$, where $L(x)=\frac{x-1}{n}$ can be considered as a private key. Internal auditors often conduct comprehensive risk assessments to learn about internal controls’ efficiency and find possible weak spots in an organization. The management team and the auditing committee should receive a written report outlining the actions of the internal audit team or department no less than once every three months. An evaluation of the system of internal controls and a recommendation section are required components of such a report.

### 3.4 Pseudocode for setting up of Paillier crypto phase

**fn** Encrypt setup phase ():

 publicKey, privateKey = generate keys()

 Initialize Blockchain ()

 deploy SmartContracts(network)

 setup *accessgranted*(*u*, *res*)

 evaluate *SC*(*reqValid*(*u*, *res*))

**return** publicKey, privateKey, network

**fn** GenerateKeys of Paillier () **do**

 Initialize large prime numbers ℘_1_, ℘_2_

 calculate *n* = ℘_1_×℘_2_

 compute $\varphi =lcm(({℘}_{1}-1)({℘}_{2}-1))$

evaluate $\mu \to L({\left({en(M}_{vl}\right)}^{\varphi }\;mod\;n^{2})$

**return** (*n*, *g*), (*φ*, *μ*)

**fn** InitializeBlockchain () **do**

**return** HyperledgerFabricSetup () with $HASH(H_{i}-1\|D\|M)$

**fn** Decrypt () **do**

compute audit_report ($de({\zeta (M}_{vl})))$

$de({\zeta (M}_{vl}))=(({\left({en(M}_{vl}\right)}^{\varphi }\;mod\;n^{2})∙\mu )mod\;n$

**Return** SC (operations, ${\left({en(M}_{vl}\right)}^{\varphi }$, network ∀ *sec*_*score*_)

**fn** deploy SC (network) **do**

deploy SC (*exp*_*loss*_)

 Compute SC, Ω(*role*(*u*))(*res*)

**return** SC

**fn** setup role-based(network) **do**

 define roles ()

 assign Permissions ()

**return** secure audit data

A secure computation and storage of encrypted audit risk values represented as *Aud*_*R*_ in the blockchain, ensuring auditability and tamper resistance. For identifying the internal audit data with inherent risk termed as *in*_*R*_ is calculated as:

$${Aud}_{R}={in}_{R}\times (1+e^{-{ctrl}_{R}})\times \log_{10}\left(1+{det}_{R}\right)$$ (7)

Eq (7) allows the multiplication of encrypted values by $1+e^{-{ctrl}_{R}}$ and application of logarithmic functions such as log_10_(1+*det*_*R*_) without decrypting individual values. The audit analysis for calculating multiple risk factors and audit parameters is a comprehensive measure to identify the *in*_*R*_ in Eq (8), are associated with transactions or audit criteria without considering controls.

$${in}_{R}=\left(\frac{{PARA}_{A}\times {Score}_{A}\times {Risk}_{A}}{{PARA}_{B}+1}\right)\times {Risk}_{B}+{Score}_{B}$$ (8)

The applied hyperledger fabric distributed ledger technology provides a safe and immutable record of transactions by encrypting the inherent risk *in*_*R*_ values. By controlling computations and decryptions, intelligent contracts may guarantee that only authorized parties can access and perform operations. Allows the safe calculation of the formula’s terms on encrypted data. It protects the privacy of critical risk metrics and audit results while they are being calculated.

The financial metric ℳ_*vl*_ based on the risk level of a particular sector *sec*_*score*_ helps assess the impact of sector-specific risks on financial values, calculated using Eq (9).

$${Adj}_{M_{vl}}=en(M_{vl})\times {\left(\frac{10}{{sec}_{Score}}\right)}^{2}$$ (9)

With the help of an additive Paillier homomorphic property, it allows the encrypted ${Adj}_{M_{vl}}$ to be securely multiplied by the square of $\left(\frac{10}{{sec}_{Score}}\right)$. Auditors modify financial measures and sector rankings without disclosing their precise numbers. Encrypts the ${Adj}_{M_{vl}}$ and stores it on the blockchain for safekeeping and auditability. By doing so, sensitive financial data can be protected, but an audit trail can also be kept open and visible. Auditors can use the adjusted financial values to assess various sectors’ economic well-being and risk exposure. Secure and efficient internal auditing is supported by encrypted computation and storage, which protects critical financial data and *sec*_*score*_ sector risk scores.

### 3.5 Designing the blockchain framework

Hyperledger Fabric is a permissioned blockchain technology used in this research to create trustworthy systems with established access controls. In these blockchain networks, nodes are individual computer systems or servers that maintain and validate the distributed ledger. The number of nodes can vary depending on the size and requirements of the network. This node can range from 1 to 10 sizes for small internal audit setups in which audit data is distributed and processed across varying districts. From this data, validation happens, and transactions are verified and consensus is maintained on the audit records’ state. Before storing internal audit data on the blockchain, encrypt it using the additive homo Paillier encryption algorithm to perform operations on encrypted data and integrate it into the blockchain architecture. The formula for Hyperledger fabric involves *f* as the endorsement function, *E* as the endorsement result, and *β* represents the response from the *i*^*th*^ endorsing peer calculated from Eq (10).

$$E=f(\beta_{1},\beta_{2},.\beta_{n})$$ (10)

They were integrating encrypted audio data into blocks with the set of audio records. With homomorphic encryption with the linking of blocks, calculations can be performed on encrypted information without deciphering it. Each block header contains a hash of the previous block’s header, creating a chain.

$$H_{i}=HASH(H_{i}-1\|D\|M)$$ (11)

Where *H*_*i*_ in Eq (11) defines the current blocks header hash, *H*_*i*_−1 denotes the previous block’s header hash, and ‖*D*‖ denotes the concatenation of block data *D*, in addition to the block metadata *M*.

Because it allows audit procedures to be handled on encrypted audit records, this is especially helpful for protecting the privacy of audited data.

Establish a blockchain network with peers, and smart contracts are developed to handle encrypted data transactions. Utilize private data collections in Hyperledger fabric to restrict access to encrypted audit data based on roles and permissions.

#### 3.5.1 Role-based access control verification in blockchain

*role*(*u*) denotes the role assigned to the user *u*, and the permission Ω denotes the permissions associated with a role. The term *accessgranted*(*u*, *res*) refers to the computation of access control module defined by the boolean function that checks if user *u* is granted access to a resource based on their role. If Ω(*role*(*u*))(*res*) returns true, access is granted; otherwise, access is denied. A Smart Contract (SC) is invoked to enforce access control rules. Let Eq (12) be computed as

accessgranted(u,res)→Ω(role(u))(res) (12)

SC(reqValid(u,res))→accessgranted(u,res) (13)

The *SC* in Eq (13) ensures that the access request parameters *u* and *res* are valid and comply with predefined conditions. If the request is valid, it triggers the *accessgranted* function to determine if access should be granted.

From Table 2, users like internal auditors, external auditors, IT administrators, audit managers and regulatory bodies are granted rights according to their assigned roles, streamlining access control through preset roles. Rules out unauthorized access by executing logic recorded on the blockchain. It checks for legitimate access requests and assigns permissions according to the user’s role and established policies.

if (role = = “internal auditor”) {grant full access;}

if (role = = “audit manager”) {grant review and management access;}

**Table 2:** Authorized parties and access control modules.

Authorized party	Attribute	Access rights
Internal auditors	*H*_*R*_	Full access
Audit managers	All attributes with review calculations	Full access
External auditors	*exp*_*loss*_ provides independent verification	Specific access
IT administrators	Blockchain infrastructure for system integrity	Enterprise infrastructure access only
Regulatory bodies	Compliance data with ${Adj}_{M_{vl}}$	Limited access

Table 3 defines the role-based access control matrix. BlockCryptoAudit’s role-based access control structure guarantees safe and proper data handling by outlining distinct rights for each position. All audit data is thoroughly managed, and internal auditors and audit management can review it. The tick mark ✓ provides the guaranteed access permission for the authorized entity, whereas the cross-symbol ✘ defines the restricted access to the corresponding entity. While IT administrators can access the blockchain system’s infrastructure for system maintenance, external auditors can access only the resources needed to do their jobs. To ensure compliance, regulatory agencies are granted restricted access. This structure prioritizes data security, integrity, and privacy, consistent with BlockCryptoAudit’s goals of enhancing audit transparency and security through blockchain and encryption technology.

**Table 3:** Role-based access control matrix.

Role	Full access	Review access	Specific access	Infrastructure access	Limited access
Internal auditors	✓	✓	✓	✘	✘
Audit managers	✓	✓	✓	✘	✘
External auditors	✘	✘	✓	✘	✘
IT administrators	✘	✘	✘	✓	✘
Regulatory bodies	✘	✘	✘	✘	✓

Integrating additive homomorphic Paillier encryption with blockchain technology allows the proposed BlockCryptoAudit technique to incorporate security considerations. Blockchain technology offers security characteristics like immutability, data privacy, and role-based access control. On the other hand, it could be considered a constraint if a thorough security study is lacking, such as assessing possible attack pathways, resilience against specific threats, or performance under different security situations. To make things clear, while the system does include robust security safeguards, it may still require a comprehensive security study or formal verification of its resilience under diverse threat models before its effectiveness can be adequately validated.

### 3.6 Developing smart contracts and audit trail automation

Smart contracts are developed to manage access control policies and user permissions. These written smart contracts automatically log all interactions with audit data, ensuring transparency and immutability. The proposal verifies that the storage, updating, and querying of encrypted audit records are all tasks that smart contracts can handle.

### 3.7 Internal audit control evaluation

The blockchain provides an unchangeable record of all computations and transactions, making them impossible to alter. Auditors can make better conclusions about risk control and management evaluations with encrypted *exp*_*loss*_ Calculations derived in Eq (14).

$${exp}_{loss}=\rho \times R_{D}\times {PARA}_{B}\times {Score}_{B}\times {Dis}_{loss}+1$$ (14)

The probability of risk events occurring within an enterprise is essential for assessing the likelihood of fraudulent activities. *R*_*D*_ associated with specific audit criteria relevant to internal audit risk assessment. *PARA*_*B*_ defines the parameter value for specific audit criteria to evaluate performance and compliance. The performance score related to particular audit criteria, *Score*_*B*_ indicating the effectiveness of audit controls in an enterprise. The financial or operational losses within a specific unit *Dis*_*loss*_, indicates the critical loss when evaluating the impact of identified risks.

As part of its enterprise audit data, continuous monitoring of the BlockCryptoAudit framework encrypts and analyzes audit data in real-time to spot suspicious trends and outliers. This procedure can quickly and efficiently resolve any operational problems or security risks. Integrating blockchain technology to generate immutable records, instantaneous encryption utilizing Paillier homomorphic encryption, and continuous data collection from several sources are crucial components. Smart contracts automate the generation of notices to enterprise individuals when predetermined performance metrics are discussed in a subsequent section, ensuring that audit messages are transmitted in a timely and secure manner.

## 4. Experimental evaluations

### 4.1 Data description

The dataset comprises detailed audit-related data with 700 + records employed to assess and manage risks within an enterprise. The data can be accessed from https://www.kaggle.com/datasets/sid321axn/audit-data?select=trial.csv [29] to conduct internal audits, prioritize audit activities, and ensure robust internal controls. There are 26 attributes utilized in this dataset; the features closely related to internal audit data are inherent risks for identifying material misstatement without any related controls. Control_risk indicates a misstatement that could occur and not be prevented and corrected by internal controls. Detection_risk represents that the auditor’s procedures will not detect fraudulent messages. Audit_risk supports the auditors can unknowingly fail to modify their opinion on financial statements that are materially misstated, and the risk factor indicates an overall representation of risk in either 0/1. Internal auditors use such in-depth risk metrics to assess the risk situation and verify that sufficient controls are in place to reduce the impact of identified risks. Table 4 defines the internal audit attributes, descriptions, and system impacts.

**Table 4:** Internal audit attributes, descriptions, and system impacts.

Attribute	Description of system impact
Sector_score (*sec*_*score*_)	Indicates the risk level of a particular sector
PARA_A/ SCORE_A/ Risk_A (*PARA*_*A*_/*Score*_*A*_/*R*_*A*_)	Parameter, score, risks for specific audit criteria *A*
PARA_B/ SCORE_B/ Risk_B (*PARA*_*B*_/*Score*_*B*_/*R*_*B*_)	Parameter, score, risks for specific audit criteria *B*
Money_value (ℳ_*vl*_)	Financial metric indicating the value of transactions
District_loss (*Dis*_*loss*_)	Losses within a specific district or enterprise operational unit
PROB (*ρ*)	Probability of risk events occurring within an enterprise.
Inherent_risk (*in*_*R*_)	The inherent risk level of transactions without considering controls (performs audit planning)
CONTROL_RISK (*ctrl*_*R*_)	A risk that controls will fail to detect fraudulent errors (access control policy, audit testing)
Detection_risk (*det*_*R*_)	Performs audit assurance impacts audit procedures.
Audit_risk	Measures overall audit effectiveness and impacts audit planning and reporting.

### 4.2 Performance comparison

The research is implemented in a Python environment using a Charm-crypto 0.50version library for evaluating the efficiency of cryptographic algorithms. An existing model like B-OAP [18], BSE-DF [23], and EG-FLB [24] contrasted with BlockCryptoAudit due to their significant advancements in blockchain-based audit systems. These models represent diverse and advanced blockchain applications in auditing, providing a robust audit setup for evaluating BlockCryptoAudit’s unique contributions. This research evaluates the framework with varying performance metrics such as risk mitigation, audit quality, security overhead, and audit trail effectiveness. These metrics can be analyzed on two conditions ranging from the number of audit operations as 100 to 700, including recording a transaction, verifying data, performing a risk assessment calculation, generating an audit report, and accessing encrypted audit records. With the implementation of nodes in blockchain interpretation for small enterprises, 1 to 10 nodes are considered for analysis.

#### 4.2.1 Risk mitigation analysis

One of the most essential elements of auditing is reducing potential dangers, especially when protecting sensitive financial information. The efficiency of the system in lowering the risks connected with data integrity, illegal access, and fraudulent actions in audit processes is measured by risk mitigation. The project aims to improve audit systems’ risk mitigation capabilities by integrating blockchain technology with advanced encryption methods, such as Paillier encryption derived using the risk mitigation score from Eq (15).

$$R_{mi}(score)=\left(1-\frac{{res}_{R}}{I_{R}}\right)\times 100$$ (15)

Where *I*_*R*_ represents untreated risk level of transactions without considering controls or mitigation measures. The remaining risk *res*_*R*_ after implementing the blockchain-based audit system and encryption measures, coupling blockchain technology’s immutability with the homomorphic characteristics of Paillier encryption improves the risk mitigation in BlockCryptoAudit. A few audit-related concerns can be efficiently mitigated by tamper-evident storage and safe computation of encrypted data.

**Fig 2:**
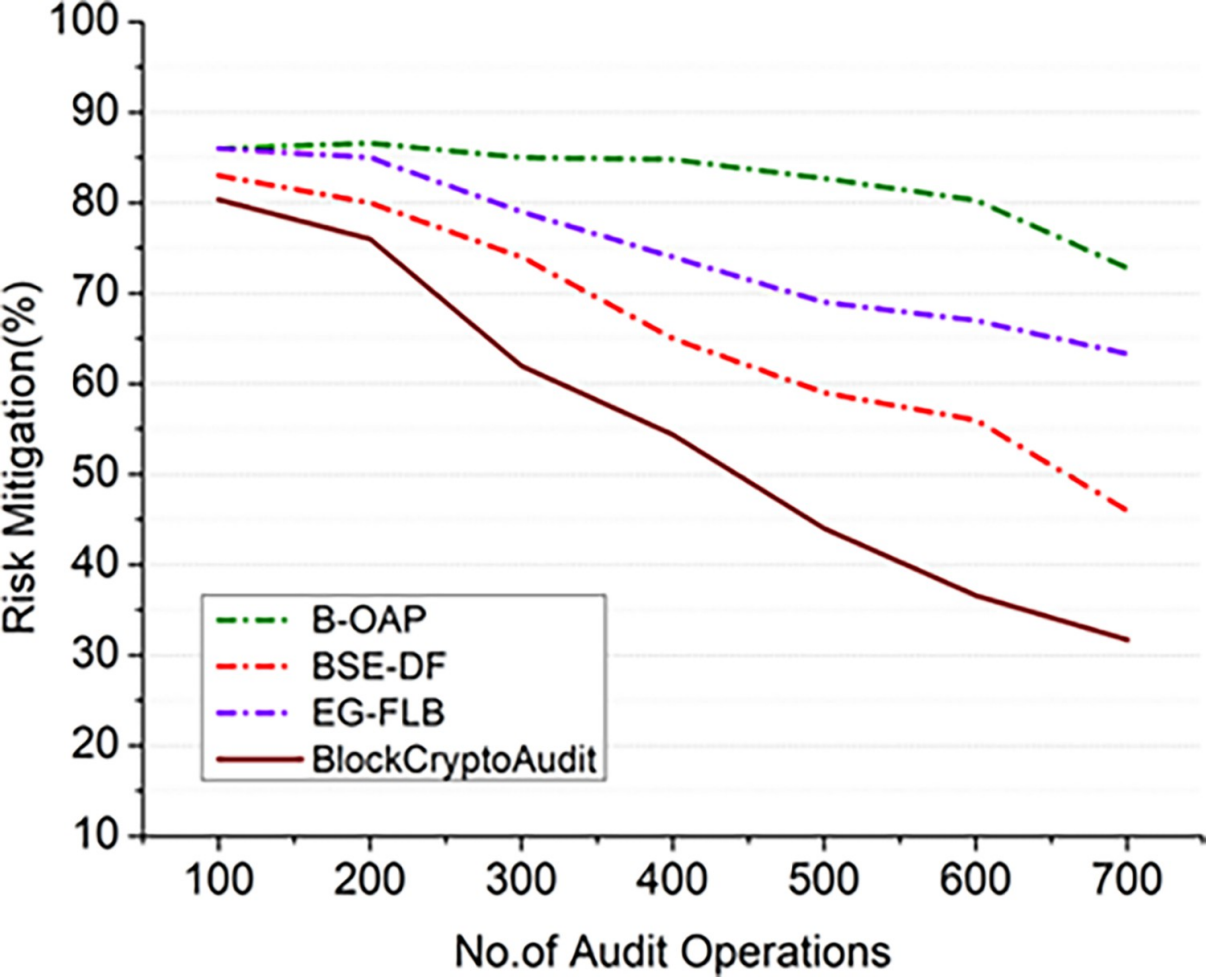
Risk mitigation comparison analysis (audit operations).

**Fig 3:**
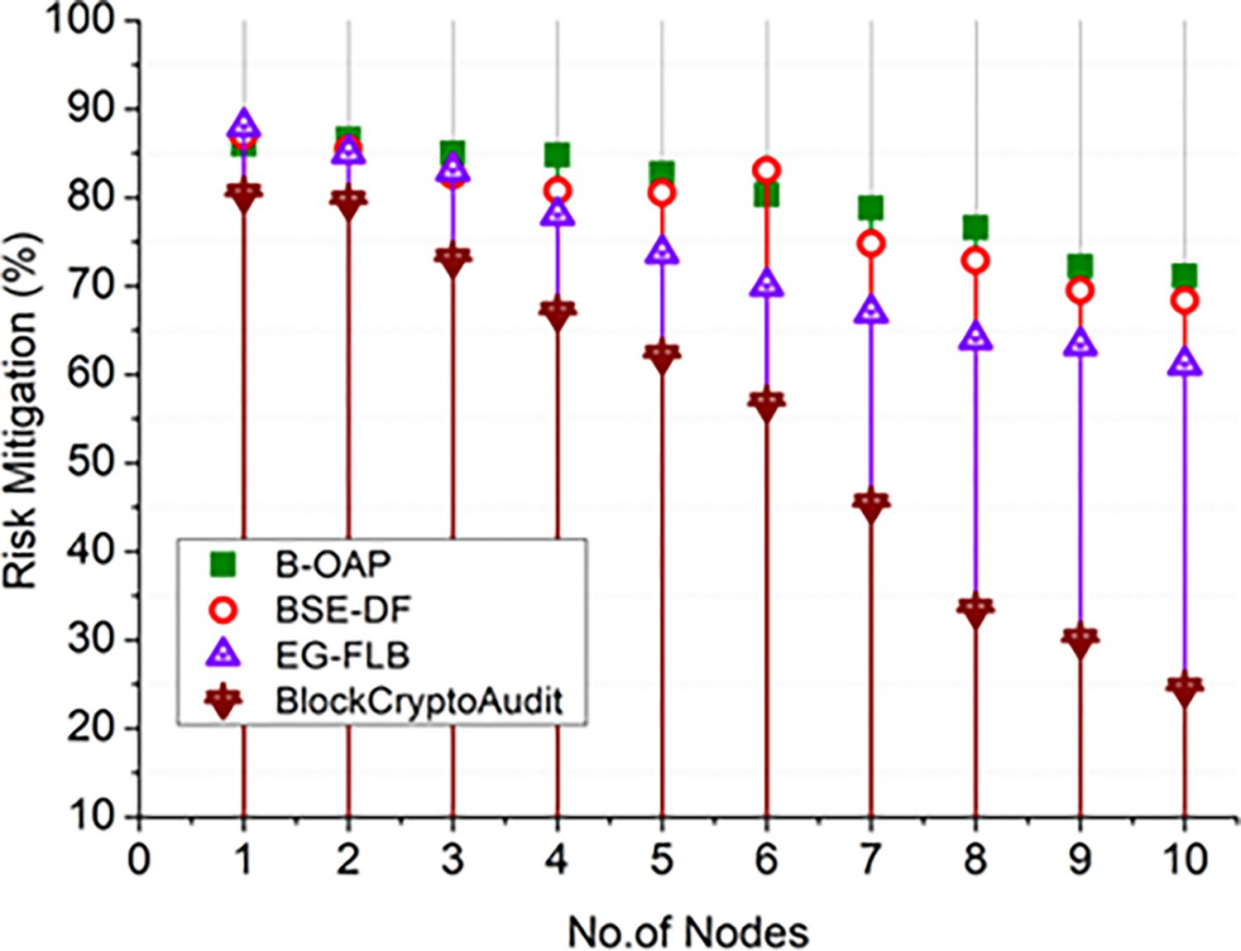
Risk mitigation comparison analysis (nodes).

The effectiveness of BlockCryptoAudit in mitigating risks is compared to older methods in Figs 2 and 3. As the number of audit operations rises, the graph shows that risk scores are determined by applying the mathematical condition $\left(1-\frac{{res}_{R}}{I_{R}}\right)$ decrease. It suggests that more thorough auditing improves risk detection. On the other hand, an increase in the number of nodes indicates that decentralization enhances security. Better risk assessment and identification are made possible with more data points, leading to better risk mitigation. Reliability and increased decentralization make risk mitigation better. All models demonstrate a declining trend in scores for risk with increasing numbers of audit procedures.

### 4.2.2 Audit trail effectiveness

This study optimizes the auditing procedure for information-based businesses by creating an internal audit process in a communication enterprise. The success of BlockCryptoAudit’s audit trail is determined by how much the system records all audit measures comprehensively, securely, and verifiable. While the blockchain creates an unchangeable record, the Paillier encryption keeps data private while enabling computations.

Audit trail effectiveness is based on Eq (16).

$$ATE=\frac{c_{A}}{t_{0}}\times D_{R}$$ (16)

Where *cA* is the comprehensive audit actions, *t*_0_ denotes the total number of audit operations and *D*_*R*_ denotes the data replication factor.

**Fig 4:**
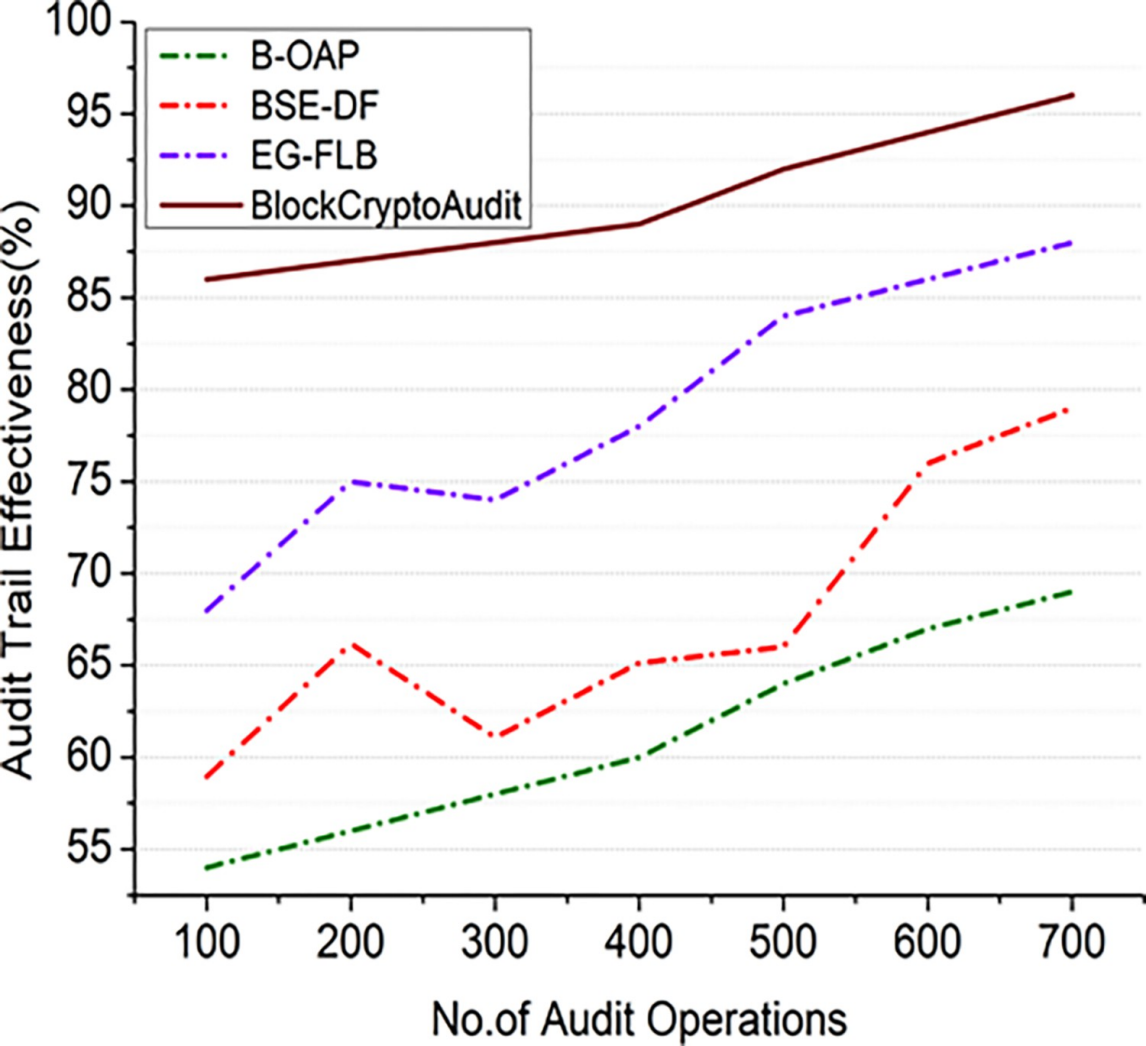
Audit trail effectiveness (audit operations).

**Fig 5:**
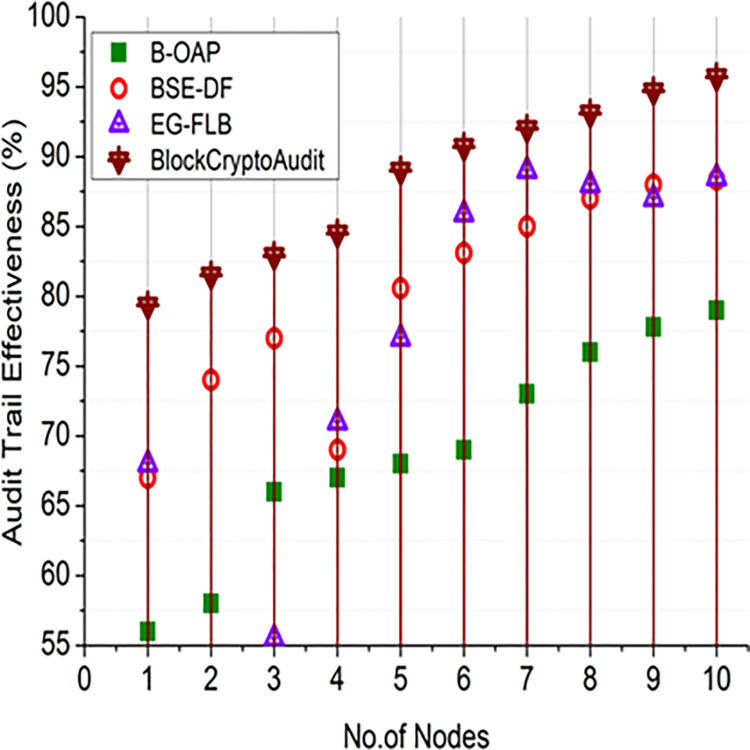
Audit trail effectiveness (nodes).

Figs 4 and 5 shows how BlockCryptoAudit’s audit trail performs compared to other models. Increasing the number of audit operations results in better efficacy, as shown in Fig 4; this indicates that more thorough auditing is being conducted. Fig 5 shows that the efficacy grows with the number of nodes, suggesting that a more distributed network has better audit trail reliability and fault tolerance. If audit actions are recorded more thoroughly, the effectiveness of the audit trail is enhanced. Improving the dissemination and replication of audit records enhances the effectiveness of the audit trail.

#### 4.2.3 Comparison of audit quality

The impact on these metrics is evaluated by altering the total number of audit operations and varying nodes performing the audit. To improve data validation and reduce errors, increasing the number of audit activities can help improve accuracy by thoroughly checking more records. On the other hand, increasing the number of nodes can help enhance data validation and encryption by the Paillier encryption system. To ensure full audits are performed across all domains, additional nodes are necessary to support decentralized access to records and analysis. Audit quality is calculated based on Eq (17).

$$Q=\frac{C_{R}\times A_{S}}{R_{M}}$$ (17)

**Fig 6:**
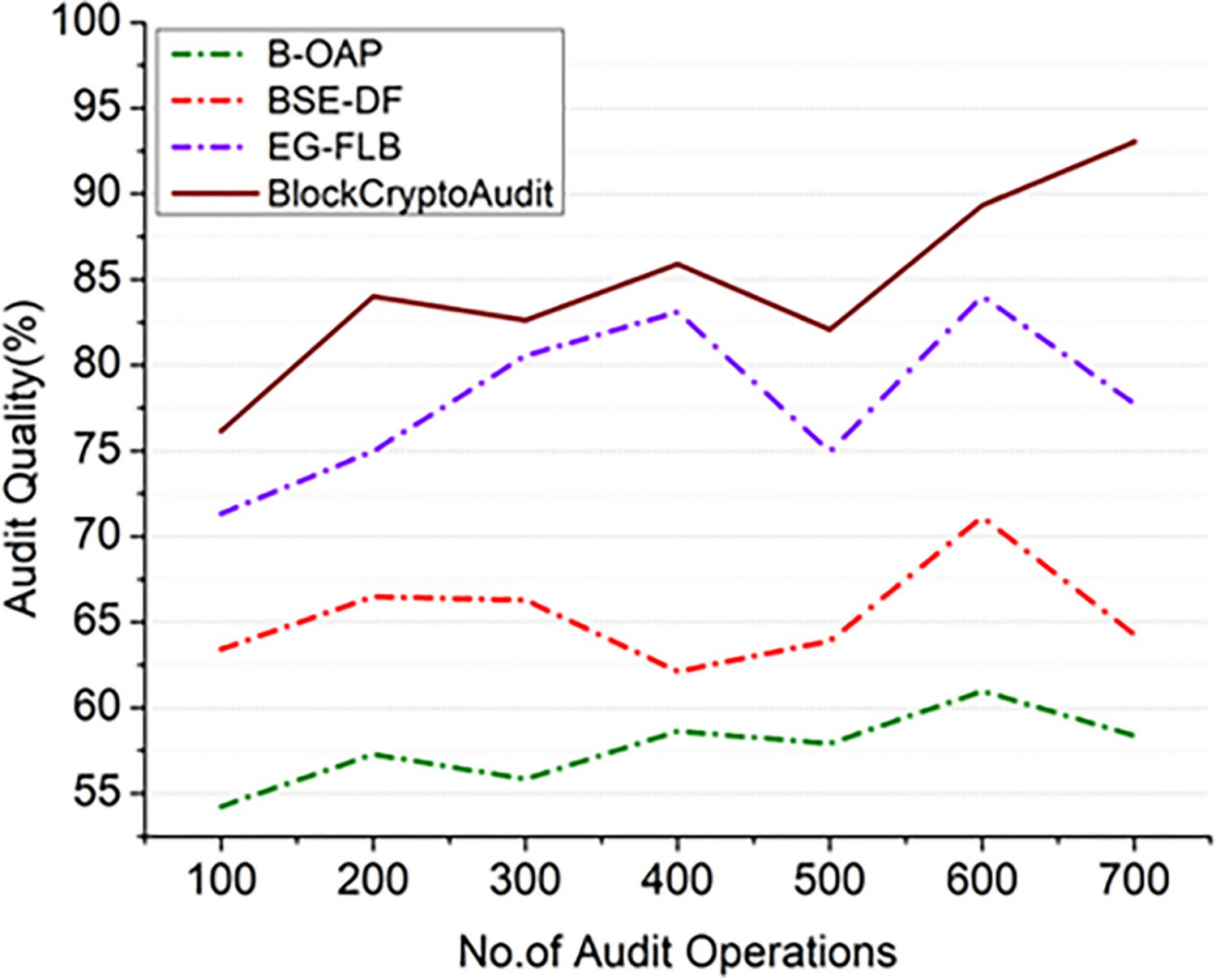
Comparision of audit quality (audit operations).

**Fig 7:**
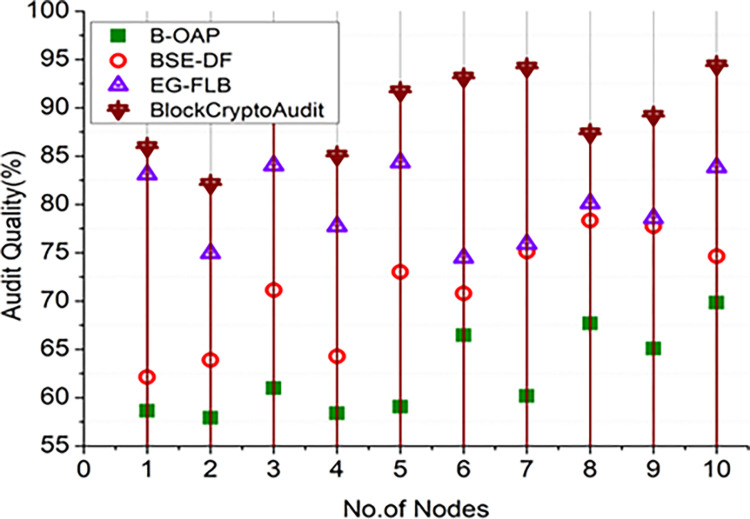
Comparision of audit quality (nodes).

Where *C*_*R*_×*A*_*S*_ is the correctness ratio in audio scope and *R*_*M*_ the Audit Risk Magnitude (the total audit risk score, including identified and unidentified concerns). A blockchain-based audit system can benefit from these differences since they assist in learning the ideal configuration for ensuring that inherent risk *in*_*R*_ with observed risk for each criterion *Score*_*A*_/*R*_*A*_ with appropriate audit quality maintained at a high level. Figs 6 and 7 illustrates the audit quality analysis on varying numbers of audit operations in Fig 6 and the number of nodes employed in the blockchain distributed ledger to maintain audit records, which are analyzed in Fig 7.

#### 4.2.4 Security overhead computation

Paillier encryption and blockchain operations significantly impact the computational expenses of the BlockCryptoAudit framework’s security overhead. Due to increased encryption/decryption processes and blockchain consensus techniques, the system incurs significant overhead as the number of audit operations or nodes increases. Security overhead is given in Eq (18).

$$SO=\frac{Computational\;cost+Blockchain\;cost}{Total\;Audit\;operations}$$ (18)

**Fig 8:**
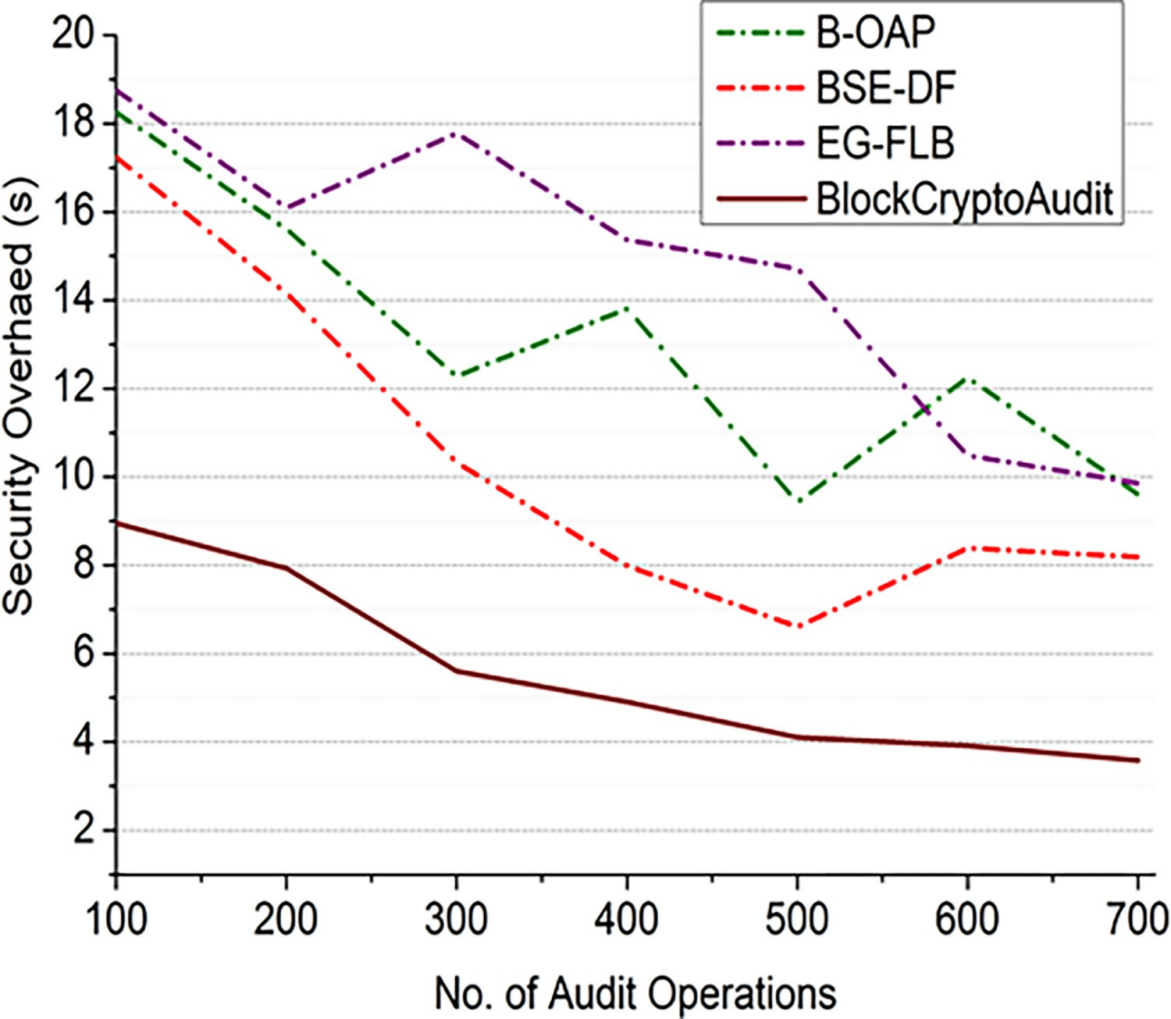
Comparison of security overhead (audit operations).

**Fig 9:**
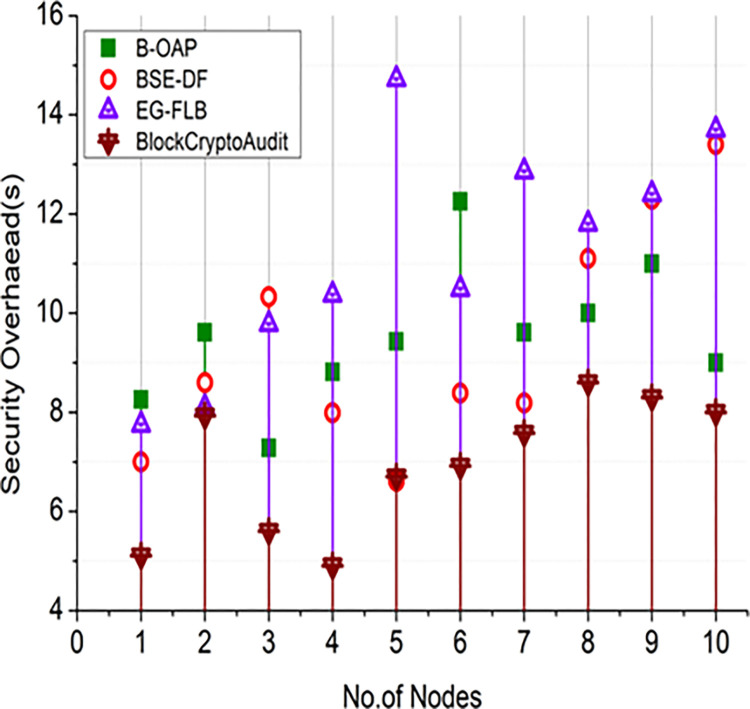
Comparison of security overhead (nodes).

For different numbers of Fig 8 audit operations and Fig 9 nodes, Figs 8 and 9 compares the security overhead of BlockCryptoAudit to that of existing models (B-OAP, BSE-DF, and EG-FLB). As the number of audit operations increases, BlockCryptoAudit shows superior scalability by showing lower growth in security overhead.

As the number of nodes in the network grows, BlockCryptoAudit’s overhead stays relatively constant, indicating that the computational load is distributed efficiently. Because blockchain and encryption are so efficient, the security overhead for BlockCryptoAudit is growing, but not as quickly. With its stable security overhead, BlockCryptoAudit demonstrates excellent scalability.

#### 4.2.5 Computation cost

The following Table provides a comparative analysis of the calculation cost of the proposed BlockCryptoAudit framework compared to other models that are already in existence, such as B-OAP, BSE-DF, and EG-FLB. In particular, this analysis demonstrates how BlockCryptoAudit manages encrypted data operations and interactions with blockchains in terms of the effectiveness of its computation. The findings should explain that the enhanced security and transparency provided by BlockCryptoAudit more than compensate for the higher processing costs resulting from integrating additive homomorphic encryption with blockchain technology. The suggested BlockCryptoAudit architecture achieves the highest possible level of computational efficiency by taking a tabular approach to evaluate the processing expenses of each model.

Table 5 shows that the proposed paradigm uses blockchain technology and additive homomorphic Paillier encryption to ensure that calculations are secure and efficient. The blockchain-based B-OAP has a moderate processing cost since it uses encryption and consensus mechanisms. This is because of the nature of blockchain. Considering the Blockchain and encryption-heavy nature of BSE-DF, processing rates are slower and compute costs are higher than expected. Regarding EG-FLB, the combination of complex encryption and blockchain integration results in significant computing overhead, ultimately leading to decreased speed. The performance and efficiency of the several models—BlockCryptoAudit, B-OAP, BSE-DF, and EG-FLB—in managing encrypted data operations and interactions with blockchain technology are the primary factors contributing to the computation cost comparisons shown in Table 5. These factors are crucial to the assertions made about computation costs:

- Computing cost of encryption techniques: The computational cost of each model’s encryption techniques is essential. Complex encryption systems like BSE-DF and EG-FLB require a lot of processing power during encryption and decryption. Hence, they have a higher computational overhead. However, lockCryptoAudit’s additive homomorphic Paillier encryption allows computations on encrypted data without decryption, reducing costs.
- Blockchain Processing Costs: How successfully the blockchain maintains consensus, validates transactions, and keeps records can affect these costs. According to the developers, BlockCryptoAudit’s optimized architecture for these operations reduces computational load compared to models that use more resource-intensive blockchain procedures.
- Scalability: Evaluation of each model’s performance as nodes and audit procedures increase is crucial. BlockCryptoAudit scales better than competing alternatives, minimizing the cost of adding nodes or audit procedures.

**Table 5:** Computation cost.

Model	Computation Cost
B-OAP	Medium
BSE-DF	High
EG-FLB	High
BlockCryptoAudit	Low

#### 4.2.6 Storage overhead

For auditing procedures, the term “storage overhead” refers to the additional space required to store audit data, encryption keys, and any associated information. This overhead can increase when dealing with blockchain technology due to the necessity of maintaining immutable records, transaction histories, and encrypted data. When compared to models that store unencrypted data or massive transaction logs, the storage optimization methods that BlockCryptoAudit implements result in a reduction in the amount of storage that is required. Organizations can conserve funds on storage costs and keep performance high with BlockCryptoAudit since its storage overhead is lower than other models. It shows how efficient it is at storing audit data. The storage overhead values for all the models are in the table. Table 6 compares BlockCryptoAudit, B-OAP, BSE-DF, and EG-FLB storage overhead. It shows that encrypted audit data, transaction records, and metadata need more space. BlockCryptoAudit’s 150 MB storage overhead is the lowest, indicating effective data management, compared to EG-FLB’s 300 MB, which demands more resources. The use of optimized encryption techniques *en*(*m*1+*m*2) derived using Eq (2) reduces the storage space required for encryption audit data. The paillier encryption scheme used in BlockCryptoAudit ensures that encrypted messages are compact, and thus minimizes the storage for encrypted transaction. The encryption of audit messages involves modular exponentiation *en*(ℳ_*vl*_), ensuring that the resulting cipher text is stored efficiently derived using an Eq (4), that contributes directly to the lower storage overhead. The lower computational and blockchain costs as derived using an *SO* from Eq (18) achieves a lower footprint compared to other existing models. Thus 150 MB storage overhead for BlockCryptoAudit is a result of its effective use of encryption, blockchain cost management, and risk-based data optimization. This allows for reduced storage needs compared to other models like EG-FLB (300 MB) and BSE-DF (257 MB), requires more resources due to less optimized techniques.

**Table 6:** Storage overhead.

Model	Storage Overhead (MB)
B-OAP	164
BSE-DF	257
EG-FLB	300
BlockCryptoAudit	150

#### 4.2.7 Energy consumption

A comprehensive evaluation of auditing procedures, including data encryption and decryption, blockchain operations, and storage management, can be done by measuring the overall energy consumed. Systems that depend on intensive computations and large data storage are particularly vulnerable to high energy consumption. Reducing the number of computations needed for encrypted data processing, BlockCryptoAudit applies optimized methods to minimize energy utilization. Values for each model’s energy usage are shown in Table 7. The auditing models’ kilowatt-hour (kWh) energy consumption is assessed in this table. When compared to B-OAP (7.5 kWh), BSE-DF (10.0 kWh), and EG-FLB (12.0 kWh), BlockCryptoAudit’s energy consumption of 5.0 kWh is the lowest, showing its superior energy efficiency. The energy consumption in BlockCryptoAudit is minimized by optimizing both the computational and blockchain costs, leading to lower energy consumption during each audit operation. From an Eq (17) the energy consumed per audit operation relative to the computational resources $\frac{C_{R}\times A_{S}}{R_{M}}$ lowers energy consumption by optimizing the relationship between these factors, and efficiently managing audit operations which contributes to the 5.0 kWh total energy consumption. These optimizations collectively contribute to the attainment of reduced energy consumption of 5.0 kWh, making BlockCryptoAudit more energy-efficient than other models like B-OAP, BSE-DF, and EG-FLB. Especially in large-scale or resource-constrained settings, this reduced energy use suggests improved performance and increased sustainability.

**Table 7:** Energy consumption.

Model	Energy Consumption (kWh)
B-OAP	7.5
BSE-DF	10.34
EG-FLB	12.12
BlockCryptoAudit	5.32

### 4.3 Practical implications

This study adds to existing knowledge on internal audit functions by integrating blockchain technology to enhance data security, authenticity, and transparency. By guaranteeing encrypted and tamper-proof auditing records, the results offer a decision-making reference for preventing corporate fraud and significantly boosting the effectiveness and reliability of internal audits in enterprises. This strategy can be an ideal precedent if other companies want to use blockchain technology to improve their internal audit procedures.

A fundamental limitation is that the framework may face challenges related to scalability with large datasets and high transaction volumes. To resolve this, future work could focus on optimizing the encryption and blockchain protocols for improved performance at scale. Additionally, incorporating machine learning techniques could enhance the automated risk assessment capabilities.

Blockchain technology may be expensive for auditing. This is primarily due to the blockchain network’s high energy and processing needs. Businesses that process plenty of data and transactions may find blockchain technology too expensive to implement and manage. Businesses that need blockchain technology may find these fees excessive. Costs include transaction fees, storage, and integrating blockchain technology with current systems. Blockchain promotes security and transparency, but smaller organizations may find the financial and resource overhead too high to adopt it extensively.

Table 8 summarizes the evaluation of BlockCryptoAudit in comparison to other models: B-OAP, BSE-DF, and EG-FLB. The assessment is based on four essential metrics: Risk Mitigation, Audit Quality, Security Overhead, and Audit Trail Effectiveness. Key findings from comparing BlockCrypto Audit to B-OAP, BSE-DF, and EG-FLB models.

**Table 8:** Performance comparison table.

Metric	Blockcrypto Audit	B-OAP	BSE-DF	EG-FLB
Risk Mitigation	High(98% mitigation)	Medium (95%)	High (90%)	High (92%)
Audit Quality	Very High (99%)	Medium (85%)	High (88%)	High (90%)
Security overhead	Low	Medium	High	High
Audit trail Effectiveness	Very High (95%)	High (88%)	High (89%)	High (91%)

BlockCrypto Audit mitigates risk better than B-OAP, BSE-DF, and EG-FLB, with a lower residual risk score as audit activities expand. Because it uses homomorphic encryption and blockchain technology, BlockCrypto Audit enhances audit data accuracy and dependability over earlier models. BlockCrypto Audit’s encryption and blockchain operations incur security overhead, but it scales better than BSE-DF and EG-FLB when nodes and audit procedures rise. BlockCrypto Audit keeps a completer and more tamper-proof audit trail than previous models, regardless of operations or nodes. The results show that BlockCrypto Audit improves internal audit procedures, specifically risk reduction and safe, transparent audit trails.

## 5. Conclusion remarks

With the integration of blockchain technology with additive homomorphic Paillier encryption, the BlockCryptoAudit framework is introduced as a novel approach to improve internal auditing. The model provides a safe and scalable system for audits, which effectively handles essential issues like data security, transparency, and the immutability of the audit trail. Evaluations comparing BlockCryptoAudit to models such as B-OAP, BSE-DF, and EG-FLB show that it reduces storage overhead and energy usage while performing better in risk mitigation, audit quality, and audit trail efficacy. These results show that the framework is much better than traditional approaches and existing blockchain-based models when securely computing and storing sensitive audit data. According to these findings, BlockCryptoAudit has excellent promise as an effective tool for internal audits in various sectors that are transparent and securely conducted.

## Supporting information

S1 File Nomenclature and equations. (DOCX)

## Abbreviations

**Notation**: **Description**
ℳ_*vl*_: Money value, representing financial transactions
R_A_,R_B_: Risk factors for audit criteria A and B
in_R_: Inherent risk level without considering controls
ctrl_R_: Control risk, indicating failure of controls to detect errors
det_R_: Detection risk, related to the auditor’s procedures
Aud_R_: Audit risk, cumulative risk of audit processes
H_R_: Historical risk based on past audit results
enm_vl_: Encrypted money value using Paillier encryption
φ: Carmichael function used in Paillier decryption
μ: Modular inverse in Paillier decryption
$R_{F_{i}}$: Risk factors associated with each historical data point
℘_1_ and ℘_2_: Large prime numbers used in key generation for encryption
H_i_: Historical data point quantifying the risk level
n: Product of large prime numbers ℘_1_ and ℘_2_
r: Random integer used in Paillier encryption
SC: Smart contract used to enforce access control
Ω: Permissions associated with user roles
E: Endorsement function in Hyperledger Fabric
