# Supplementary material for: Enterprise internal audit data encryption based on blockchain technology
Source: PLoS One. 2025 Jan 10;20(1):e0315759. doi: 10.1371/journal.pone.0315759 (PMC11723644; doi:10.1371/journal.pone.0315759)
Supplement: S1 File — (DOCX) [file pone.0315759.s001.docx]

**EQUATIONS**

| Eq. (1) | $H_{R}=\sum_{i=1}^{n} \left( \frac{H_{i},}{max(H)} \right)^{2}\times R_{F_{i}}$ |
| --- | --- |
| Eq. (2) | $en\left( m1+m2 \right)=en\left( m1 \right)\cdot en\left( m2 \right) mod n^{2}$ |
| Eq. (3) | ${n=℘}_{1}{\times℘}_{2}$ |
| Eq. (4) | $en\left( \mathcal{M}_{vl} \right)=g^{m_{vl}}\cdot r^{n} mod n^{2}$ |
| Eq. (5) | $de\left( {\zeta\mathcal{(M}}_{vl}) \right)=\left( \left( {({en\mathcal{(M}}_{vl})}^{\varphi} mod n^{2} \right)\cdot\mu\right) mod n$ |
| Eq. (6) | $\varphi=lcm(\left( ℘_{1}-1 \right)\left( ℘_{2}-1 \right))$ |
| Eq. (7) | ${Aud}_{R}={in}_{R}\times\left( 1+e^{-{ctrl}_{R}} \right)\times\log_{10} \left( 1+{det}_{R} \right)$ |
| Eq. (8) | ${in}_{R}=\left( \frac{{PARA}_{A}\times{Score}_{A}\times{Risk}_{A}}{{PARA}_{B}+1} \right)\times{Risk}_{B}+{Score}_{B}$ |
| Eq. (9) | ${Adj}_{\mathcal{M}_{vl}}=en\left( \mathcal{M}_{vl} \right)\times\left( \frac{10}{{sec}_{Score}} \right)^{2}$ |
| Eq. (10) | $E=f(\beta_{1},\beta_{2},.\beta_{n})$ |
| Eq. (11) | $H_{i}=HASH\left( H_{i}-1\left\Vert D \right\Vert M \right)$ |
| Eq. (12) | $accessgranted(u, res)\to\Omega\left( role(u) \right)\left( res \right)$ |
| Eq. (13) | $SC(reqValid\left( u,res \right))\to accessgranted(u, res)$ |
| Eq. (14) | ${exp}_{loss}=\rho\times R_{D}\times{PARA}_{B}\times{Score}_{B}\times{Dis}_{loss}+1$ |
| Eq. (15) | $R_{mi}\left( score \right)=\left( 1-\frac{{res}_{R}}{I_{R}} \right)\times100$ |
| Eq. (16) | $ATE=\frac{c_{A}}{t_{0}}\times D_{R}$ |
| Eq. (17) | $Q=\frac{C_{R}\times A_{S}}{R_{M}}$ |
| Eq. (18) | $SO=\frac{Computational cost+Blockchain cost}{Total Audit operations}$ |
